# Inflammasome Priming Is Similar for *Francisella* Species That Differentially Induce Inflammasome Activation

**DOI:** 10.1371/journal.pone.0127278

**Published:** 2015-05-18

**Authors:** Mohammed G. Ghonime, Srabani Mitra, Ramadan A. Eldomany, Mark D. Wewers, Mikhail A. Gavrilin

**Affiliations:** 1 Davis Heart & Lung Research Institute and Pulmonary Allergy Critical Care and Sleep Medicine Division, The Ohio State University, Columbus, OH, 43210, United States of America; 2 Microbiology and Immunology Department, Faculty of Pharmacy, Helwan University, Cairo, Egypt; University of California Merced, UNITED STATES

## Abstract

Inflammasome activation is a two-step process where step one, priming, prepares the inflammasome for its subsequent activation, by step two. Classically step one can be induced by LPS priming followed by step two, high dose ATP. Furthermore, when IL-18 processing is used as the inflammasome readout, priming occurs before new protein synthesis. In this context, how intracellular pathogens such as *Francisella* activate the inflammasome is incompletely understood, particularly regarding the relative importance of priming versus activation steps. To better understand these events we compared *Francisella* strains that differ in virulence and ability to induce inflammasome activation for their relative effects on step one vs. step two. When using the rapid priming model, i.e., 30 min priming by live or heat killed *Francisella* strains (step 1), followed by ATP (step 2), we found no difference in IL-18 release, p20 caspase-1 release and ASC oligomerization between *Francisella* strains (*F*. *novicida*, *F*. *holarctica* –LVS and *F*. *tularensis* Schu S4). This priming is fast, independent of bacteria viability, internalization and phagosome escape, but requires TLR2-mediated ERK phosphorylation. In contrast to their efficient priming capacity, *Francisella* strains LVS and Schu S4 were impaired in inflammasome triggering compared to *F*. *novicida*. Thus, observed differences in inflammasome activation by *F*. *novicida*, LVS and Schu S4 depend not on differences in priming but rather on their propensity to trigger the primed inflammasome.

## Introduction

Pathogen associated molecular patterns (PAMP) of infectious agents are recognized through a set of germ-line encoded pattern recognition receptors (PRR). Nod-like receptors (NLRs) represent a family of the most widely studied PRRs because of their role as intracellular sensors and their ability to form inflammasomes. The assembly of the multi-protein complex, inflammasome, is required for the activation of caspase-1 which processes the proinflammatory cytokines proIL-1β and proIL-18 into their mature forms. In contrast to IL-1β which requires stimulation and hours for its synthesis, IL-18 is constitutively present in monocytes and can be immediately released after cell stimulation leading to inflammasome activation [1]. IL-18 plays a major role in modulating the subsequent adaptive immune responses [2, 3]. It is well established that IL-18 together with IL-12 and IL-15 drive the T_H_1 mediated adaptive immune response, primarily inducing interferon gamma production in NK and T cells [4, 5]. Thus, early release of active IL-18 provides an important step in combating infectious pathogens.

Caspase-1 activation by the inflammasome platform, intensively studied for NLRP3, requires 2 steps: step 1—priming and step 2—activation or triggering. Our recent report showed that priming is a rapid process that occurs within minutes of LPS exposure and is necessary for licensing subsequent inflammasome activation [6]. Although the rapid two step mechanism of inflammasome activation is well documented for endotoxin (step 1—LPS priming and step 2—ATP- dependent P2X7 receptors activation), it is not clear how intracellular bacteria activate inflammasome function in the two-step process. In this context, it was recently shown that a biphasic activation mechanism is needed for the Nlrc4 inflammasome where Ser533 phosphorylation primes Nlrc4 for subsequent activation by the flagellin sensor Naip5 [7]. To study the mechanisms of inflammasome activation by intracellular bacteria, we used several strains of *Francisella* which differ in their virulence and ability to induce inflammatory response. *Francisella tularensis* is a facultative intracellular Gram-negative bacterium that causes tularemia. There are four subspecies of *Francisella*: *F*. *tularensis* (Type A, includes Schu S4 strain), *F*. *holarctica* (Type B, includes live vaccine strain—LVS*)*, *F*. *mediasiatica* and *F*. *novicida*. *F*. *tularensis* (Schu S4) is highly virulent as less than 10 colony forming units can initiate disease in human and mice [8, 9]. In contrast, *F*. *holarctica* is more virulent for mice than for humans whereas *F*. *novicida* while being highly virulent for mice is almost completely avirulent for humans [8]. Of note, avirulent *F*. *novicida* can infect human monocytes and induces a more robust inflammatory response as compared to virulent *F*. *tularensis* and *F*. *holarctica* [10–13].

Macrophage infection by *Francisella* is characterized by a multifaceted lifecycle that is essential for its pathogenesis. It begins with initial bacteria recognition at the cell membrane [14] followed by incorporation into a *Francisella*-containing phagosome (FCP) with subsequent escape into the cytosol [10, 15, 16] where it undergoes extensive replication inducing inflammasome activation and macrophage pyroptosis [16–19]. In the cytosol, intracellular *Francisella* subspecies can be recognized by intracellular PRRs including AIM2 [20, 21], pyrin [22, 23] and NLRP3 [24, 25] resulting in inflammasome activation and IL-1β/IL-18 release. However, when utilizing IL-1β processing as the readout, this process requires hours to allow detection of visible inflammasome activation. ProIL-1β synthesis is the time limiting factor. In contrast, another inflammasome substrate, proIL-18, eliminates this time constraint. IL-18 is constitutively present in unstimulated monocytes thus allowing the study of priming of the inflammasome as a distinct event in caspase-1 activation. We have recently used IL-18 processing as a signature of early inflammasome activation to focus on the factors that regulate inflammasome priming by LPS [6, 26]. In the present work we utilized several *Francisella* species differing in inflammasome activation (step 2) as physiological infectious models to study how *Francisella* species diverge in inflammasome priming and activation phases. We show that different *Francisella* species can prime inflammasome activation to a similar degree in freshly isolated monocytes. Although the *Francisella* priming effect is TLR2 mediated, it is also ERK-dependent like we have shown for LPS priming [6]. Importantly, in the absence of ATP stimulation, *Francisella* internalization is required to provide the final activation (i.e., step 2).

It is widely discussed that *F*. *tularensis* Schu S4 and LVS can escape detection by the immune system and induce no or weak immune response. Here we show that priming, signal 1 in inflammasome activation, is similar for all species of *Francisella*, live and dead, equally licensing inflammasome for the second signal. Thus, observed differences in inflammasome activation by various strains of *Francisella* likely depend on their ability to avoid or suppress triggering of step 2.

## Material and Methods

### Cell culture, bacterial strains and infections

Human PBMCs were isolated by Histopaque density gradients from fresh source leukocytes from the American Red Cross. Monocytes were isolated from PBMC by CD14 positive selection (MACS, Miltenyi Biotec, Auburn,CA) as we describe elsewhere [6]. This method of purification yields greater than 98% pure monocytes based on flow cytometry analysis. Monocytes (1x10^6^/ml) were incubated in culture tubes in RPMI 1640 (MediaTech, Inc, Manassas, VA) supplemented with 10% heat-inactivated FBS (Atlas Biologicals, Fort Collins, CO) in the absence of antibiotics. FBS lots were prescreened for endotoxin contamination to confirm that they did not induce IL-18 release by ATP in the absence of LPS. *F*. *novicida* U112 (JSG1819) and it *iglC* and *mglA* mutants, *F*. *holarctica*-LVS and *F*. *tularensis* Schu S4 were generously provided by Dr. John Gunn (The Ohio State University) and grown on chocolate II agar (BD Biosciences, Sparks, MD) at 37°C, harvested, and resuspended in cell culture medium before adding to cells to calculate multiplicity of infection (MOI). Heat killed *Francisella* (HK) was prepared by heating at 95°C for 10 min. Killed bacteria suspension was plated on chocolate II agar plates to ensure effective killing. All experiments involving *F*. *tularensis* Schu S4 were performed in a BSL3 facility at The Ohio State University as previously described [13].The first step, priming, was initiated by monocyte incubation with bacteria or various TLR ligands for 30 min. We used LPS from *Escherichia coli* strain 0111:B4 (Alexis Biochemicals, San Diego, CA), highly purified LPS from *F*. *novicida* and *F*. *tularensis*-LVS (gift from John Gunn), Pam3CysSK4 (EMD Millipore, Billerica, MA), Malp2 (Novus Biological, Littleton, CO), Poly(I:C) and CpG (InvivoGen, San Diego, CA). TLR2 and TLR4 signaling was blocked by CU CPT 22 (Tocris Bioscience, UK) and RS-LPS from *Rhodobacter sphaeroides* (InvivoGen, San Diego, CA). Phagocytosis was blocked by actin polymerization inhibitors cytochalasin D (Sigma-Aldrich, St. Louis, MO) and latrunculin (Cayman Chemical, Ann Arbor, MI). The second step, inflammasome activation, was induced by ATP (5 mM) from Sigma-Aldrich for 30 min as well. Potassium efflux was blocked by media supplemented with 100 mM of KCl (Sigma-Aldrich). ERK inhibitor UO126 was purchased from (EMD Millipore, Billerica, MA), tyrosine kinase inhibitor AG126 and ATP-sensitive potassium channel blocker glybenclamide were from InvivoGen (San Diego, CA), In parallel experiments, monocytes were incubated with *Francisella* for 2 h to allow bacteria internalization, then gentamycin was added (10 μg/ml) to kill non-internalized bacteria and cells were incubated overnight (total incubation time 16 h). Cells then were harvested, separated from bacteria by low-speed centrifugation at 200g for 5 min; and lysed in TRIzol (Ambion, Life Technologies, Carlsbad, CA) or hypotonic lysis buffer for RNA or protein isolation, respectively. After low-speed centrifugation, cell culture media was cleared from bacteria by high speed centrifugation at 16,000g for 5 min, filtered and used for cytokine determination.

### RNA isolation and RT-qPCR

Monocytes were lysed in TRIzol reagent and stored at -80°C prior to RNA isolation. Total RNA was isolated according to the manufacturer’s recommendations and 1–2 μg of total RNA was converted to the first strand cDNA by the Thermoscript RT System (Invitrogen, Life Technologies, Carlsbad, CA) using poly-dT primer. Gene expression was quantified by real time PCR with Power SYBR Green PCR Master Mix (Applied Biosystems, Warrington, UK) in the StepOne Real Time PCR System (Applied Biosystems) and expressed in relative copy numbers (RCN) as we describe elsewhere [10]. Briefly, RCN = 2^-ΔCt^ x 100, with ΔCt calculated by subtracting the average Ct of two housekeeping controls (CAP-1 and GAPDH) from the experimental sample Ct.

### Preparation of cell lysates and Western blotting

Cells were lysed in RIPA buffer (50 mM Tris-HCl (pH7.5), 150 mM NaCl, 1 mM EDTA, 1mM NaF, 1% NP-40 and 0.25% Na-deoxycholate) supplemented with complete protease inhibitor cocktail (Sigma-Aldrich), 1 mM PMSF and 100 μM N-(methoxysuccinyl)-Ala-Ala-Pro-Val chloromethyl ketone—CMK). The protein concentrations were determined using Bio-Rad Dc protein Lowry assay (Bio-Rad, Hercules, CA). After SDS-PAGE gel electrophoresis, separated proteins were transferred to a PVDF (polyvinylidine fluoride) transfer membrane, probed with the antibody of interest, followed by HRP conjugated secondary antibody and developed by ECL (GE Healthcare, NJ) using autoradiography. Rabbit polyclonal antisera against IL-1β, ASC and caspase-1 were developed in our laboratory. Anti-human IL-18 antibody was purchased from MBL (Woburn, MA) and anti-actin from MP Biomedicals (Solon, OH). Rabbit anti-phospho-ERK1/2 and rabbit anti-ERK1/2 were purchased from Cell Signaling Tech (Danvers, MA). Released IL-1β was quantified using a sandwich ELISA format as previously reported [27] but substituting monoclonal (MAB601) from R&D Systems (Minneapolis, MN) for the capture antibody. In addition, IL-1β in the cell culture medium was detected by immunoblot of cell culture medium with rabbit polyclonal IL-1β antibody (lab generated). Released IL-18 was quantified by ELISA using MBL antibodies (Woburn, MA).

### Determination of bacterial CFU and intracellular growth

Human monocytes (10^6^/well) were infected with *Francisella* and left for the specified time. At 2 h post infection 10 μg/ml gentamicin was added to kill extracellular bacteria. Then cell culture media was collected and gently spun down (200g for 5 min) to separate cells from bacteria. Cells were washed 3 times with sterile PBS and pelleted by gentle centrifugation at 200g for 5 min and then lysed on ice with 1 ml of sodium deoxycholate for 10 min followed by repeated pipetting to complete cell lysis. Serial dilutions of the lysates were rapidly plated onto chocolate II agar plates, and plates were incubated overnight at 37°C before enumeration of CFUs. The number of viable intracellular bacteria was determined for each condition, and at least 3 independent experiments were performed.

### ASC oligomerization detection

ASC oligomerization was detected as previously reported [28]. Human monocytes were cultured in polypropylene tubes and treated with different stimuli. The cells were pelleted by centrifugation at 200g for 5 min and resuspended in 500 μl of ice-cold buffer containing 20 mM HEPES-KOH, pH 7.5, 150 mM KCl, 1% Nonidet P-40, 0.1mM PMSF and a protease inhibitor mixture, and lysed by shearing through a 21-gauge needle. The cell lysates were then centrifuged at 5,000g for 10 min at 4°C, and the resultant pellets were washed twice with PBS and resuspended in 500 μl of PBS. Next, the resuspended pellets were crosslinked with freshly prepared DSS for 30 min and pelleted by centrifugation at 5,000g for 10 min. The crosslinked pellets were resuspended in 25 μl of SDS sample buffer separated using 10% SDS-PAGE and immunoblotted using anti-human ASC antibody.

### Ethics statement

The procedure to isolate monocytes from blood samples of healthy donors and the respective consent forms were approved by the Institutional Review Board for human subject research at The Ohio State University (IRB protocol number 2011H0059). All healthy donors provided written consent for the collection of samples and subsequent analysis.

### Statistical analysis

All experiments were performed a minimum of three independent times and expressed as mean values ± SEM. Comparison of groups for statistical difference were done using Student’s *t* test. P value ≤ 0.05 was considered significant.

## Results

### *Francisella* activates inflammasome via a two-step process

The focus of this study was to determine whether differences in activation of the inflammasome by virulent vs avirulent strains of *Francisella* depend on step one (priming) or step two (activation or triggering). To isolate the priming effect of *Francisella* from the triggering step we challenged human monocytes with different *Francisella* strains for 30 min followed by a short ATP pulse for another 30 min. In contrast, overnight bacterial challenge in the absence of exogenous ATP served to test both priming and activation steps by *Francisella*.

**Fig 1:**
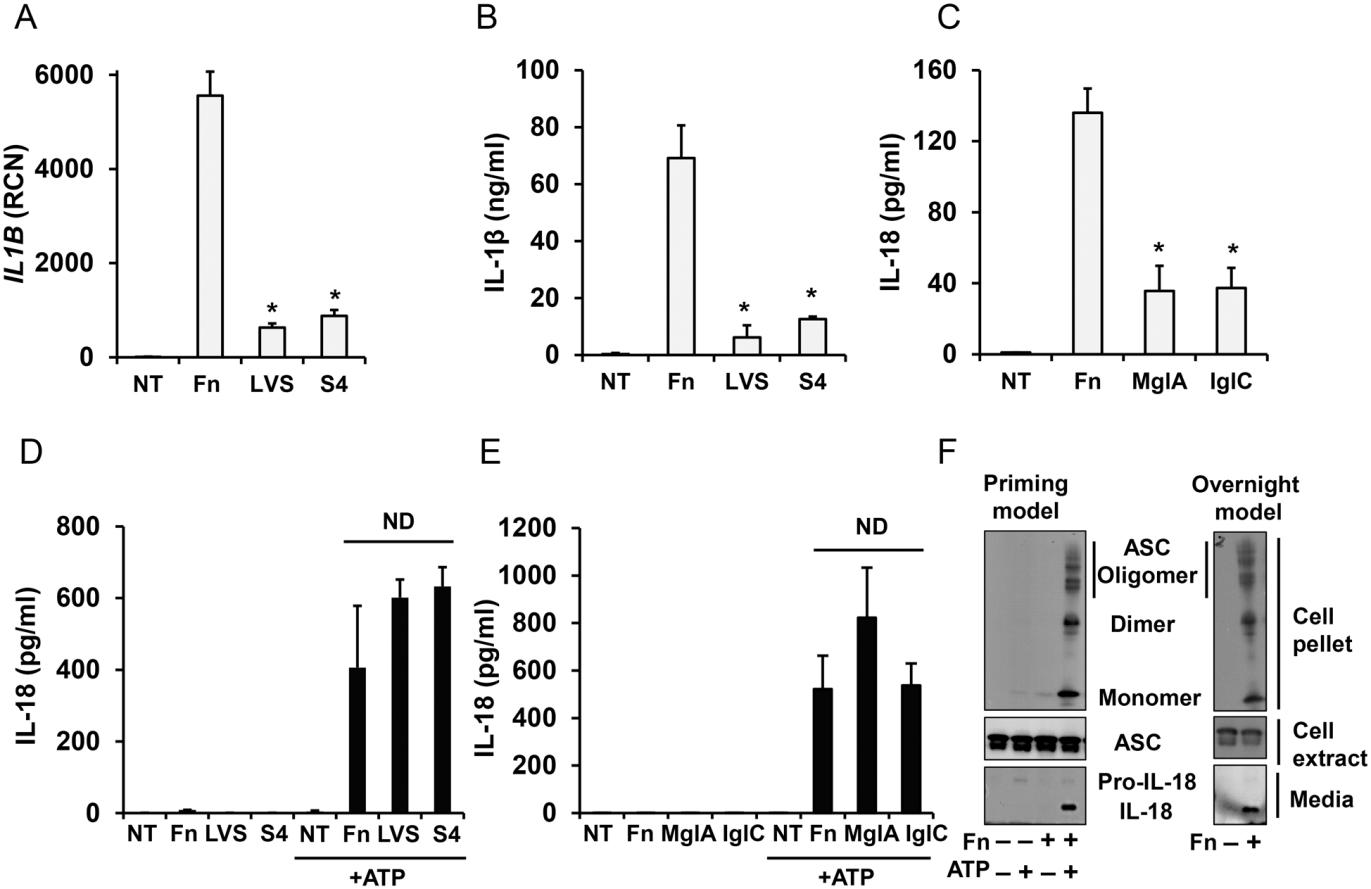
Difference in inflammasome activation by *Francisella* between rapid and overnight models. Overnight model; white bars. Primary human monocytes were left untreated (NT) or infected with 100 MOI of different strains of *Francisella* for 16 h and *IL1B* mRNA (**A**), mature IL-1β (**B**) and IL-18 (**C**) release in response to *F*. *novicida* (Fn), *F*. *tularensis*-LVS (LVS), SchuS4 (S4) and *F*. *novicida* FPI mutants *mglA* and *iglC* was measured. Priming model; black bars. When monocytes were infected with *Francisella* for only 30 min, a second signal (5 mM ATP for 30 min) was required to induce IL-18 processing and release. NT—not infected by *Francisella* (**D, E**). Monocytes stimulated with *F*. *novicida* in the priming model or overnight model were tested for IL-18 release by immunoblot and for ASC in cell lysates and oligomeric ASC in the insoluble cell pellet by ASC immunoblot (**F**). Data represent mean ± SEM, n ≥ 3 independent experiments. * p<0.05. ND—not statistically different. Blots are representative of repeated experiments.

First, we confirmed that in an overnight infection model *F*. *novicida* induces a more robust inflammasome response as compared to SchuS4 and *F*. *tularensis*-LVS (Fig 1A and 1B). To examine the priming capacity of the *Francisella* subspecies, freshly-isolated human monocytes were incubated with *F*. *novicida*, SchuS4 or *F*. *tularensis*-LVS for 30 min and then subsequently activated with ATP for 30 min. Unexpectedly, we found that all the subspecies were able to prime the inflammasome similarly as determined by the release of processed IL-18 (Fig 1D).

*Francisella* carrying mutations in the *Francisella* pathogenicity island (FPI) are impaired in phagosomal escape and intracellular growth. In keeping with phagosomal escape as crucial to step 2 in inflammasome activation, all FPI mutant *Francisella* showed reduced IL-18 processing in an overnight infection model (Fig 1C). In contrast, short term priming with *F*. *novicida* and its FPI mutants did not affect the priming capacity of these bacteria for subsequent ATP activation (Fig 1E). Finally, ASC oligomerization, as a signature of inflammasome activation, was similar between short-term priming plus ATP stimulation and long time infection models (Fig 1F).

### *Francisella* priming (step 1) is TLR2 dependent

**Fig 2:**
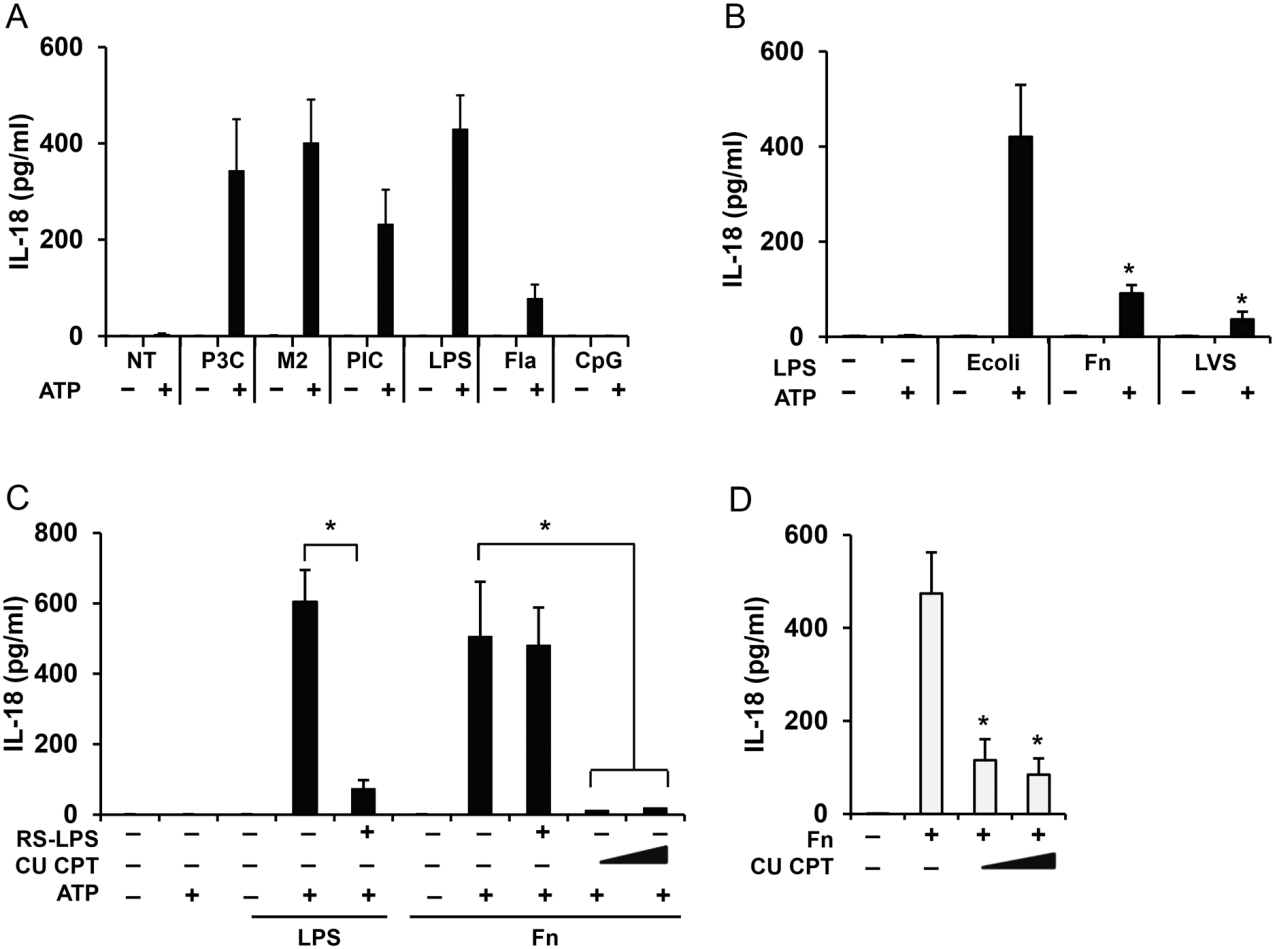
*Francisella* priming inflammasome is TLR2 dependent. Human monocytes were left untreated (NT) or primed for 30 min with TLR2, 4, 5, 6, 9 ligands: Pam3CysSK4 (P3C) (100 ng/ml), Malp2 (M2) (100 ng/ml), PolyI:C (PIC) (10 μg/ml), *E*. *coli* endotoxin (LPS) (1 μg/ml), flagellin (Fla) (100 ng/ml) or CpG (10 μg/ml), and then stimulated with ATP (5 mM) for additional 30 min and IL-18 release was measured by ELISA (**A**). IL-18 release from human monocytes primed for 30 min with 1 μg/ml of LPS from *E*. *coli*, *F*. *novicida* (Fn) and *F*. *holarctica*-LVS (LVS) and stimulated with ATP (5 mM) for 30 min (**B**). IL-18 release by monocytes, pretreated with TLR2/1 inhibitor CU-CPT (5 and 10 μM) or TLR4 inhibitor RS-LPS (1 μg/ml) for 30 min and then primed with *F*. *novicida* (Fn) for 30 min and activated with ATP (5 mM) for 30 min (**C**). IL-18 release by monocytes preloaded for 30 min with (5 and 10 μM) CU-CPT and incubated with *F*. *novicida* (Fn) for 16 h (**D**). Data represent mean ± SEM, n = 3 independent experiments. * p<0.05. White bars—overnight model, black bars—rapid priming model.

When using LPS as a TLR4 ligand, monocytes are primed within minutes for ATP induced inflammasome triggering [6]. However, *Francisella* ssp LPS has a unique structure and is unable to fully activate TLR4 [29, 30]. Instead, TLR2 is the major TLR recognizing *Francisella* [31–33]. To evaluate the ability of diverse TLR ligands to induce the priming step in inflammasome activation, we used Pam3CysSK4, Malp2, Poly(I:C), LPS, flagellin and CpG-DNA as TLR2/1, TLR2/6, TLR3, TLR4, TLR5 and TLR9 agonists, respectively. We found that all TLR agonists, except TLR9, efficiently prime the inflammasome (Fig 2A). TLR9’s inability to prime the inflammasome agrees with the recognition that the *TLR9* expression in humans is restricted to plasmacytoid dendritic cells and B cells [34, 35].

To clearly determine the ability of *Francisella* LPS to initiate priming, we compared the priming effect of LPS from *E*.*coli*, *F*. *novicida* and *F*. *holarctica*-LVS. Only *E*. *coli* LPS was able to prime monocytes while *Francisella* LPS was a weak priming agent (Fig 2B). To further determine which TLR is responsible for *Francisella* priming, monocytes were pretreated with either LPS from *Rhodobacter sphaeroides* (RS-LPS) or CU CPT as TLR4 and TLR2/1 antagonists, respectively. As shown Fig 2C, RS-LPS was able to block the *E*. *coli* LPS priming effect but not *Francisella* mediated priming, thus excluding *Francisella’s* LPS as a priming factor. In contrast, pretreatment with the TLR2 antagonist CU CPT blocked *Francisella* priming indicating a critical role for TLR2 in early sensing of *Francisella* infections. Importantly, inhibition of TLR2 signaling also led to decreased IL-18 processing and release in the overnight model which provides both step 1 and step 2 (Fig 2D).

### Inflammasome priming but not activation is independent of *Francisella* internalization

**Fig 3:**
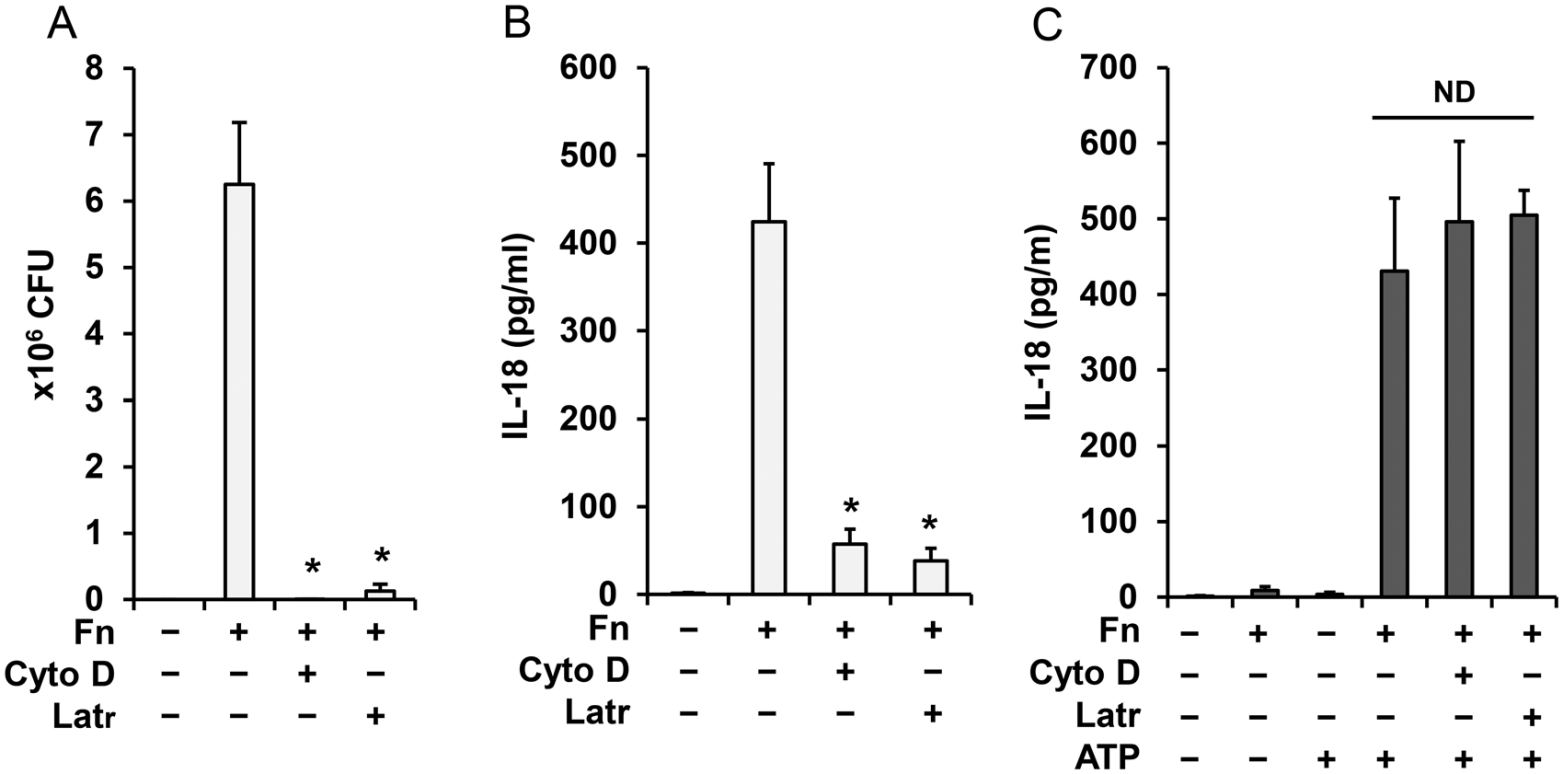
Inflammasome priming by *Francisella* is independent of bacteria internalization. Human monocytes were pretreated for 30 min with latrunculin A (Latr) (200 nM) and cytochalasin D (Cyto D) (5 μg/ml) and infected with *F*. *novicida* for 16 h. CFU of internalized bacteria (**A**) and IL-18 release (**B**) were counted. Monocytes treated with latrunculin and cytochalasin D as in A, B and then primed with *Francisella* for 30 min followed by ATP (5mM) for 30 min were analyzed for IL-18 in cell culture media by ELISA (**C**). Data represent mean ± SEM, n = 3 independent experiments. * p<0.05. White bars—overnight model, black bars—rapid priming model. ND—not statistically different.

Internalization and phagosomal escape of *Francisella* are critical for inflammasome activation and IL-1β induction, processing and release [10, 16]. On the other hand, processing and release of mature caspase-1 and IL-18 is rapid and may not require bacterial uptake. To address this point, we blocked phagocytosis with latrunculin A and cytochalasin D prior to monocyte infection with *Francisella*. These agents were able to block phagocytosis (Fig 3A) and inflammasome activation (Fig 3B) in the overnight model but they did not affect inflammasome priming by *Francisella* for the second signal—ATP (Fig 3C). In addition, the time course of *Francisella* internalization confirmed minimal uptake at early time points (S1 Fig). This finding suggests that inflammasome priming by *Francisella* occurs at the cell membrane while a combination of priming and internalization is required for *Francisella*-mediated activation of the inflammasome.

### *Francisella* infection induces ASC oligomerization in both rapid priming and overnight models, which is regulated by K^+^ efflux

**Fig 4:**
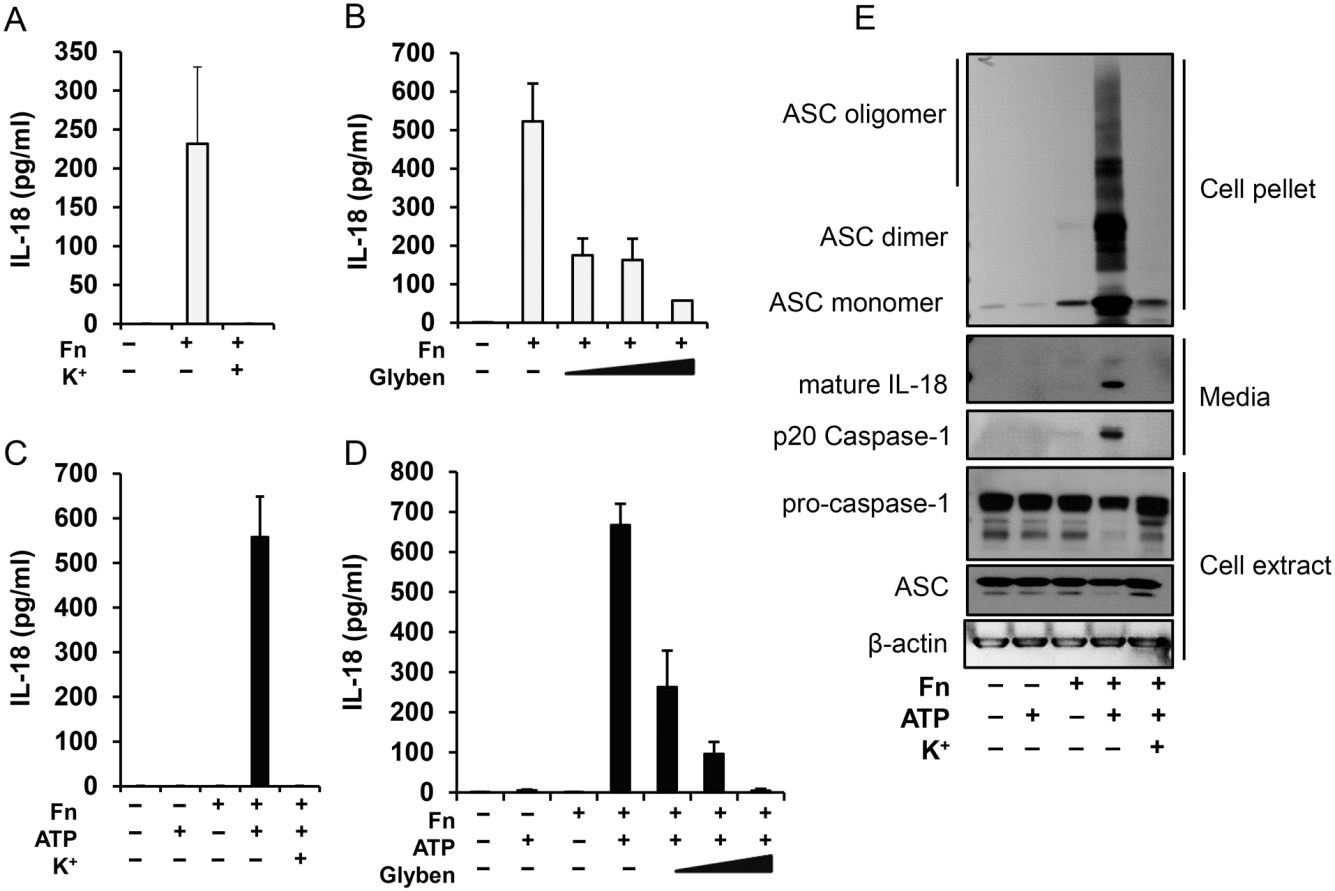
*Francisella* priming is dependent on K+ efflux. Human monocytes were pretreated with K+ (100mM) or glybenclamide (50, 100 and 200 μM) for 30 min and then infected with *F*. *novicida*. IL-18 release was measured either 16 h post-infection (**A, B**) or 30 min followed by 30 min of ATP stimulation (**C,D**). Cell culture media, extract and pellet were immunoblotted for IL-18, caspase-1 and ASC (**E**). Data represent mean ± SEM, n = 3 independent experiments. * p<0.05. White bars—overnight model, black bars—rapid priming model.

The human monocyte inflammasome complex that responds to the LPS/ATP stimulus has been previously linked to NLRP3 [6, 26]. To evaluate this relationship in the *Francisella* priming model and the overnight model we blocked K^+^ efflux with either extracellular K^+^ or with glybenclamide pretreatment, approaches that have been shown to prevent NLRP3 function [36–38]. As shown, in the overnight *Francisella* model (Fig 4A and 4B) and in the priming model with ATP (Fig 4C and 4D), both *Francisella* models depend upon K^+^ efflux to promote caspase-1 activation. In support of this finding, K^+^ blocked the formation of ASC oligomers and the release of mature IL-18 and p20 caspase-1 (Fig 4E).

### *Francisella* priming is ERK, ROS and tyrosine kinase dependent

**Fig 5:**
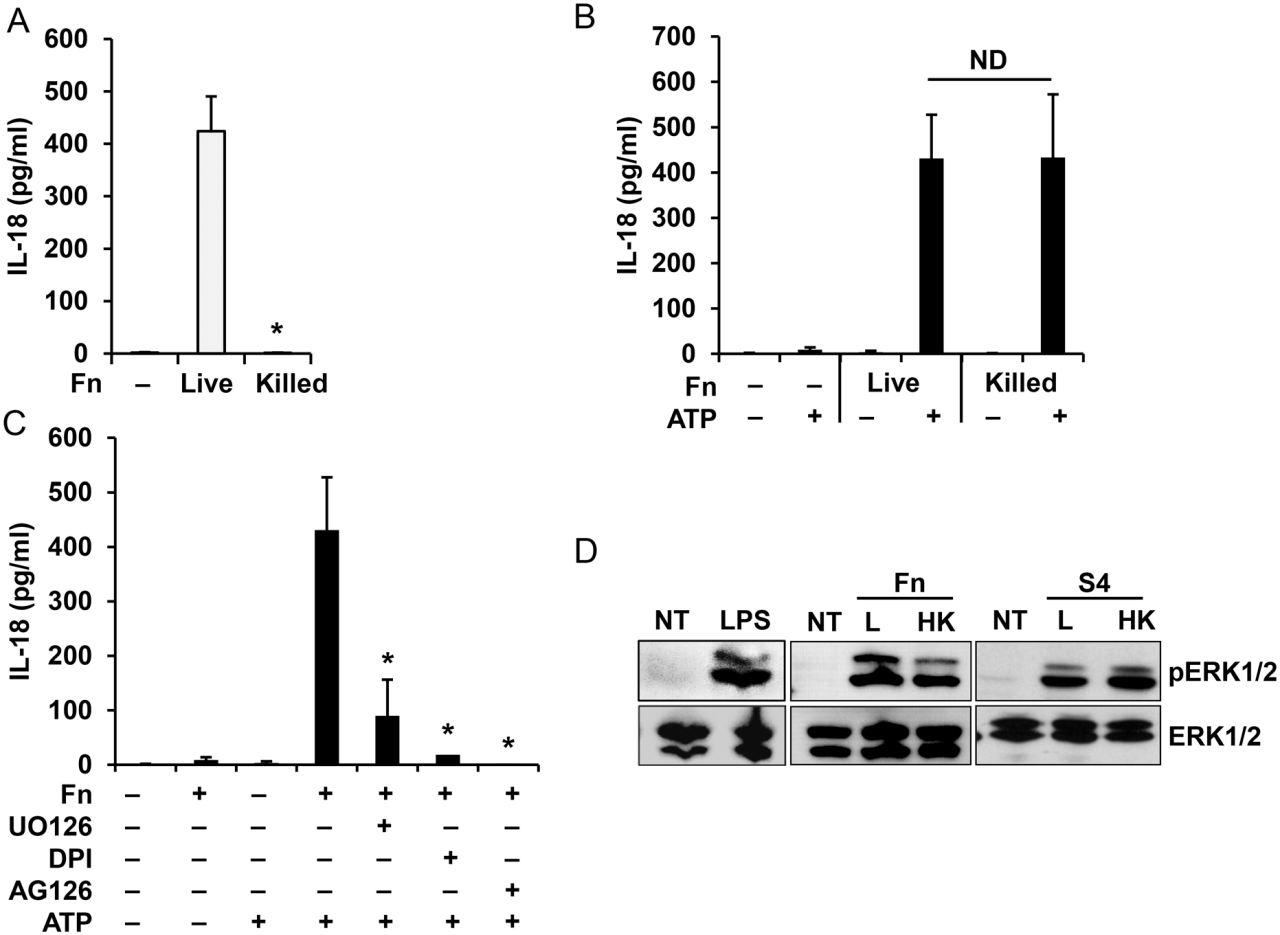
Monocyte priming by *Francisella* is ERK dependent. Human monocytes were infected for 16 h with live or killed *F*. *novicida* (Fn) and IL-18 release was measured by ELISA (**A**). Human monocytes were primed with live or killed *F*. *novicida* for 30 min followed by ATP stimulation for 30 min and release of IL-18 was measured by ELISA (**B**). IL-18 release from monocytes pretreated with UO126 (20 μM), AG126 (10 μM) or DPI (50 μM) for 30 min and then primed with *F*. *novicida* (Fn) for 30 min followed by ATP stimulation for 30 min (**C**). Monocytes, primed with live (L) or heat killed (HK) *F*. *novicida* (Fn) and *F*. *tularensis* SchuS4 (S4) for 30 min were lysed and cell lysate was analyzed for ERK phosphorylation. LPS from *E*. *coli* was used as a positive control (**D**). Data represent mean ± SEM, n = 3 independent experiments. * p<0.05. ND—not statistically different. Blots are representative of repeated experiments. White bars—overnight model, black bars—rapid priming model.

To further characterize the source of the priming ability of *Francisella*, we found that only live *Francisella* induce IL-18 processing and release in the overnight model (Fig 5A) suggesting that cytosol escape requires an active principle. However, both live and heat-killed *Francisella* can prime the inflammasome for ATP activation (Fig 5B). Using the chemical inhibitors: UO126 (ERK inhibitor), AG126 (tyrosine kinase inhibitor) and DPI (NADPH oxidase inhibitor), we showed that rapid monocyte priming by *Francisella* is dependent on ERK, ROS and a tyrosine kinase as it has been previously shown for *E*. *coli* LPS (Fig 5C) [6]. These results are in agreement with our recent work showing that the early priming of inflammasome requires ERK pathway activation [6]. In addition, both live and killed *F*. *novicida* priming can activate ERK phosphorylation (Fig 5D). These results suggest that viability of the bacteria is not required for inflammasome priming.

## Discussion

It is now recognized that inflammasome activation is a two-step process where priming is a necessary first step to prepare the inflammasome for triggering substrate cleavage in step two. This two-step process is best illustrated for the NLRP3 inflammasome by numerous studies that have used the combination of endotoxin (LPS) and ATP as inducers of proIL-1β and proIL18 processing and release [6, 26, 36, 39, 40]. In mononuclear phagocytes, low dose LPS alone does not activate IL-1β and IL-18 processing and release but requires the activation of P2X7 receptors by exogenous ATP to complete the activation of the NLRP3 inflammasome. We have recently demonstrated that LPS priming is rapid and independent of new protein synthesis when using constitutive IL-18 as the inflammasome readout in a rapid sequence LPS/ATP model [6].

*Francisella* infection appears to model the priming and activation events similar to described for LPS and ATP. However, there is a major difference in inflammasome preparedness for subsequent activation between fast priming (30 min) and classical infectious (4–16 h) models. As we have previously shown, live or killed *F*. *novicida* infection for 8–16 h can induce proIL-1β mRNA and protein synthesis [10, 13]. Yet, this protein and mRNA induction is alone insufficient to induce IL-1β processing and release since *Francisella* internalization and phagosome escape is required to supply signal 2 [10]. In contrast, in the fast priming model new mRNA and protein synthesis is not required as proIL18, pro-caspase-1 and ASC are constitutively expressed and activated when ATP is used as signal 2 [6, 26]. Using the IL-18 model we present evidence that *Francisella* can rapidly prime monocytes for immediate inflammasome activation via P2X7 receptors. This priming does not involve new RNA and protein synthesis, depends on TLR2, does not require live bacteria and is independent of bacteria internalization and phagosomal escape.

By comparing classical infectious (overnight incubation) and the fast priming models we were able to shed light on difference in inflammasome activation by several species of *Francisella*. It is well documented that in classical infectious models (4–16 h) avirulent *F*. *novicida* induces a more robust proinflammatory response than virulent *F*. *tularensis* strains. Murine macrophages and dendritic cells infected with *F*. *tularensis* Schu S4 secrete very low levels of the inflammasome-dependent cytokines IL-18 and IL-1β accompanied by weak activation of caspase-1 [25, 41, 42]. Likewise, *F*. *holarctica* LVS induces weak activation of the inflammasome in the murine macrophage [25]. Human mononuclear cells also secrete less proinflammatory cytokines in response to virulent *F*. *tularensis* SchuS4 as compared to avirulent *F*. *novicida* [13, 43]. The mechanisms of this subversion are not clear with evidence for both stealth and active suppression. Moreover, in support of the active suppression hypothesis it was shown in a co-infection model that *F*. *tularensis* Schu S4 actively suppresses proinflammatory response induced by *F*. *novicida* in human monocytes [13] and dendritic cells [44]. In addition, lipids isolated from virulent *F*. *tularensis* inhibit pulmonary inflammation in a mouse model [45]. In this study we also confirmed that monocytes infected with *F*. *novicida* release IL-1β more robustly as compared to LVS and Schu S4.

In contrast to these studies, our rapid priming model shows that all strains of *Francisella*, virulent and attenuated, equally prepare the inflammasome for activation by ATP. This priming does not require *Francisella* internalization and phagosome escape. As we show, a variety of factors that stimulate TLRs may result in similar preparedness of the inflammasome for subsequent activation by ATP. Thus, priming appears to be a critical event in inflammasome activation. In this context, *Francisella* primes the monocyte inflammasome via TLR2, in agreement with published data that *Francisella* mediates monocyte activation via TLR2 [31, 32, 46–49]. Inhibition of TLR2 by CU CPT decreased IL-18 release in both overnight and rapid priming models, where signal 2 was provided by intracellular *Francisella* or by ATP, respectively. This suggests that initial *Francisella* recognition by TLR2 is sufficient to program the inflammasome for its subsequent activation. In the absence of *Francisella* internalization, signal 2 can be provided by exogenous danger-associated molecule pattern ATP via the P2X7 receptors.

Interestingly, inflammasome activation was dependent on K^+^ efflux in both overnight infection and fast priming models using *F*. *novicida*. K^+^ efflux is the common trigger of NLRP3inflammasome activation [37]. It was previously shown that the NLRP3 inflammasome is activated in response to toxins and ATP [50]. Taking into account that there is no *Francisella* internalization in the rapid priming model (S1 Fig) and that ATP is required to complete inflammasome activation, we may conclude that rapid priming by *Francisella*, similar to LPS and other TLR ligands, prepares NLRP3 for the signal 2. Of note, inhibition of K^+^ efflux also blocked monocyte IL-18 release in response to overnight *Francisella* infection, where *Francisella* internalization is required to provide signal 2. As it is known that AIM2 and pyrin may serve as intracellular sensors of *Francisella* [21–23, 51–53], and that human but not mouse NLRP3 may be involved in cell response to *Francisella* [24], the connection of these PRR with NLRP3 and with K^+^ efflux remains to be clarified.

Lastly, similar to our previous report with LPS/ATP [6], priming by *Francisella* is dependent on ROS, tyrosine kinase and ERK phosphorylation. Moreover, activation of ERK promotes *Francisella* internalization [54] and similar ERK phosphorylation by *F*. *novicida* and Schu S4 observed in our present work is in agreement with the equal bacterial uptake reported for these two strains [13, 42]. Therefore, the observed differences in the overnight inflammasome activation model between *F*. *novicida*, LVS and Schu S4 are likely due to step two, intracellular recognition by PRR. In addition, differential activation of inflammasome (signal 2) by *F*. *novicida* and *F*. *tularensis* Schu S4 may also be predetermined by differential sensitivity and resistance of each strain to ROS [42].

In summary, inflammasome activation appears to behave as a binary weapon system in which at least two independent steps are needed to activate this critical inflammatory event. In the case of *Francisella* species we have demonstrated that step one of this process, priming, is provided by TLR2 stimulation which is not greatly different between pathogenic and nonpathogenic strains. However, step two is modulated differently between poor inflammasome activators (*Francisella* Schu S4 and LVS) and strong activators (*Francisella novicida*). Future studies directed at step two modulation by cytosolic bacteria are likely to advance our understanding of pathogen host interactions.

## Supporting Information

S1 Fig Time course of *F*. *novicida* internalization. Human monocytes were infected with *F*. *novicid*a for varying time points with and without ATP as labeled, washed to remove extracellular bacteria, lysed and then plated on chocolate II agar to calculate CFU of internalized bacteria.
(TIF)

S2 Fig Inflammasome priming and activation is independent of *Francisella* MOI. Human monocytes were infected with a range of *F*. *novicida* (MOI 20, 40, 60, 80, 100) for 30 min and then treated or not with 5 mM of ATP for another 30 min. Cell culture media was collected and IL-18 release was measured by ELISA.
(TIF)
