# Supplementary figures and images for: Inflammasome Priming Is Similar for Francisella Species That Differentially Induce Inflammasome Activation
Source: PLoS One. 2015 May 18;10(5):e0127278. doi: 10.1371/journal.pone.0127278 (PMC4436270; doi:10.1371/journal.pone.0127278)

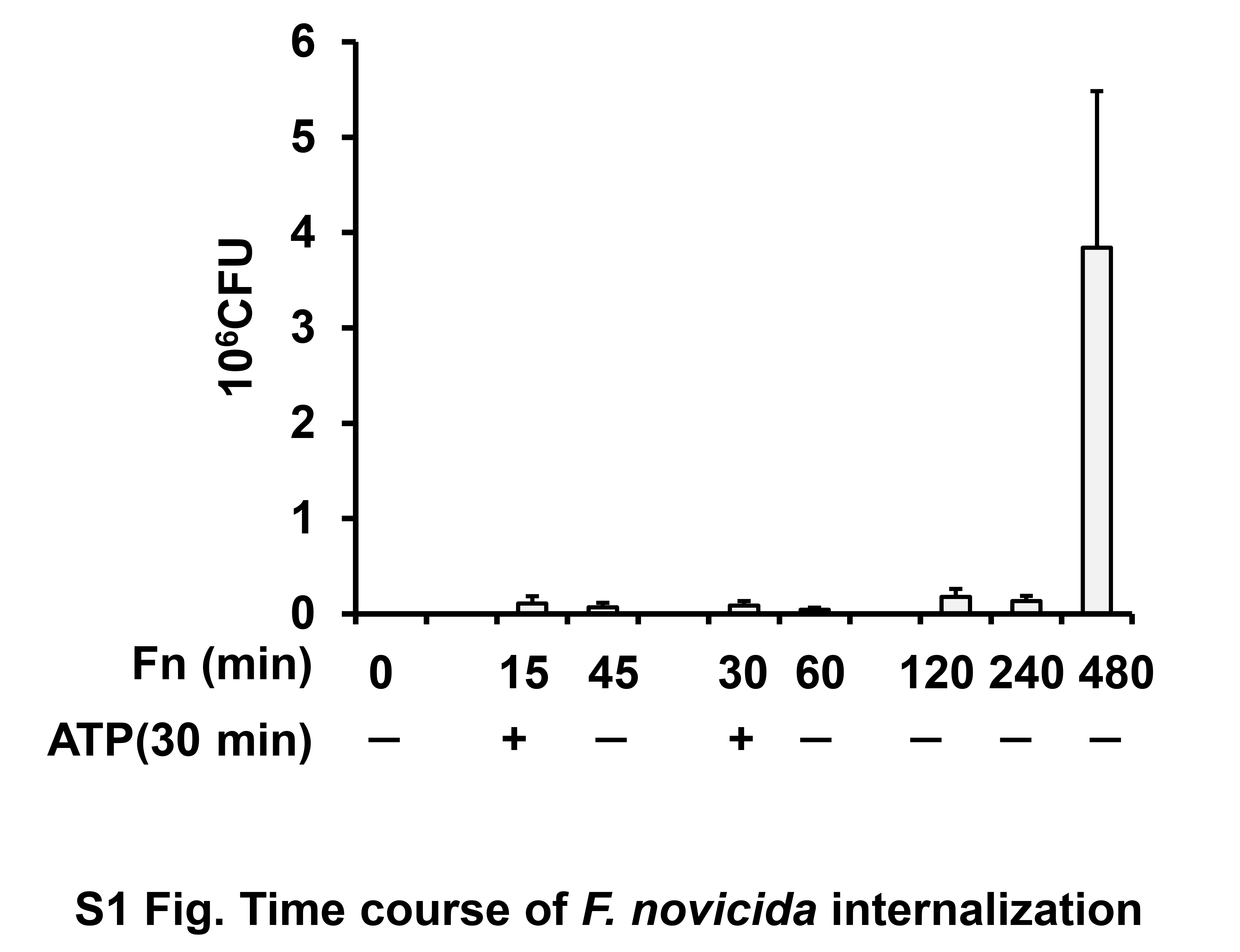

Supplement: S1 Fig — Human monocytes were infected with F. novicida for varying time points with and without ATP as labeled, washed to remove extracellular bacteria, lysed and then plated on chocolate II agar to calculate CFU of internalized bacteria. (TIF) [file pone.0127278.s001.tif]

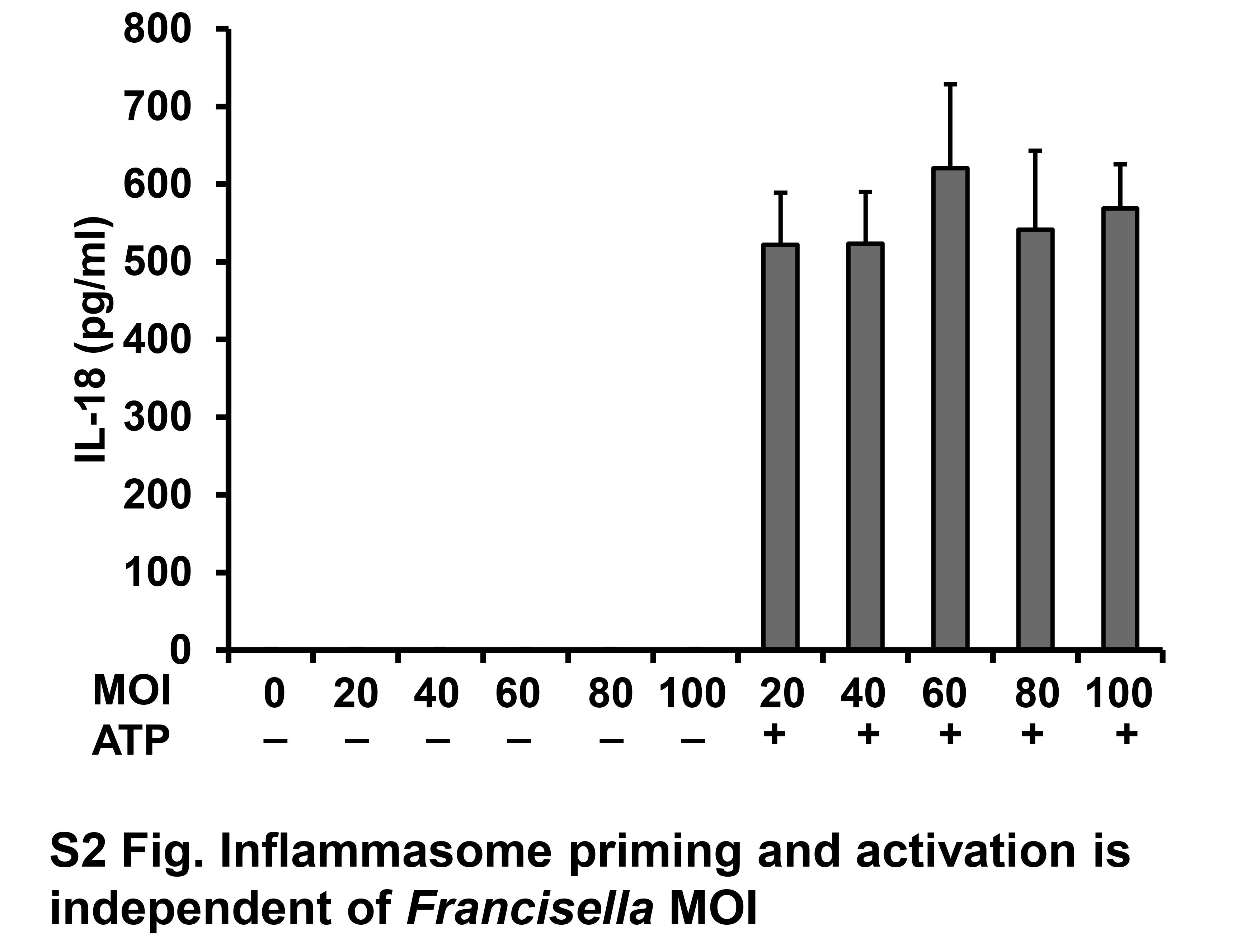

Supplement: S2 Fig — Human monocytes were infected with a range of F. novicida (MOI 20, 40, 60, 80, 100) for 30 min and then treated or not with 5 mM of ATP for another 30 min. Cell culture media was collected and IL-18 release was measured by ELISA. (TIF) [file pone.0127278.s002.tif]
